# Childhood Socioeconomic Position and Objectively Measured Physical Capability Levels in Adulthood: A Systematic Review and Meta-Analysis

**DOI:** 10.1371/journal.pone.0015564

**Published:** 2011-01-26

**Authors:** Kate Birnie, Rachel Cooper, Richard M. Martin, Diana Kuh, Avan Aihie Sayer, Beatriz E. Alvarado, Antony Bayer, Kaare Christensen, Sung-il Cho, Cyrus Cooper, Janie Corley, Leone Craig, Ian J. Deary, Panayotes Demakakos, Shah Ebrahim, John Gallacher, Alan J. Gow, David Gunnell, Steven Haas, Tomas Hemmingsson, Hazel Inskip, Soong-nang Jang, Kenya Noronha, Merete Osler, Alberto Palloni, Finn Rasmussen, Brigitte Santos-Eggimann, Jacques Spagnoli, John Starr, Andrew Steptoe, Holly Syddall, Per Tynelius, David Weir, Lawrence J. Whalley, Maria Victoria Zunzunegui, Yoav Ben-Shlomo, Rebecca Hardy, on behalf of the HALCyon study team

**Affiliations:** 1 Department of Social Medicine, University of Bristol, Bristol, United Kingdom; 2 Medical Research Council Unit for Lifelong Health and Ageing and Division of Population Health, University College London, London, United Kingdom; 3 Medical Research Council Lifecourse Epidemiology Unit, University of Southampton, Southampton, United Kingdom; 4 Department of Community Health and Epidemiology, Queen's University, Kingston, Canada; 5 Department of Primary Care and Public Health, Centre for Health Sciences Research, School of Medicine, Cardiff University, Cardiff, United Kingdom; 6 The Danish Twin Registry and The Danish Aging Research Center, Institute of Public Health, University of Southern Denmark, Odense, Denmark; 7 Department of Epidemiology, School of Public Health and Institute of Health and Environment, Seoul National University, Seoul, Republic of Korea; 8 Medical Research Council Lifecourse Epidemiology Unit, University of Southampton, Southampton, United Kingdom; 9 National Institute for Health and Research Musculoskeletal Biomedical Research Unit, University of Oxford, Oxford, United Kingdom; 10 Department of Psychology and Centre for Cognitive Ageing and Cognitive Epidemiology, University of Edinburgh, Edinburgh, United Kingdom; 11 Institute of Applied Health Sciences and Rowett Institute of Nutrition and Health, University of Aberdeen, Foresterhill, Aberdeen, United Kingdom; 12 Department of Epidemiology and Public Health, University College London, London, United Kingdom; 13 Department of Non-Communicable Disease Epidemiology, London School of Hygiene & Tropical Medicine, London, United Kingdom; 14 School of Social and Family Dynamics, Arizona State University, Tempe, Arizona, United States of America; 15 Division of Occupational and Environmental Medicine, Department of Public Health Sciences, Karolinska Institutet, Stockholm, Sweden; 16 Department of Society, Human Development and Health, Harvard School of Public Health, Boston, Massachusetts, United States of America; 17 Economics Department, Federal University of Minas Gerais, Belo Horizonte, Brazil; 18 Research Center for Prevention and Health, Glostrup University Hospital, Glostrup, Denmark; 19 Center for Demography and Ecology, University of Wisconsin, Madison, Wisconsin, United States of America; 20 Department of Public Health Sciences, Karolinska Institute, Stockholm, Sweden; 21 Institute of Social and Preventive Medicine, University Hospital Center and University of Lausanne, Lausanne, Switzerland; 22 Department of Geriatric Medicine and Centre for Cognitive Ageing and Cognitive Epidemiology, University of Edinburgh, Edinburgh, United Kingdom; 23 Institute for Social Research, University of Michigan, Ann Arbor, Michigan, United States of America; 24 Institute of Applied Health Sciences, University of Aberdeen, Aberdeen, United Kingdom; 25 Departement de Medecine Sociale et Preventive, Universite de Montreal, Montreal, Canada; Indiana University, United States

## Abstract

**Background:**

Grip strength, walking speed, chair rising and standing balance time are objective measures of physical capability that characterise current health and predict survival in older populations. Socioeconomic position (SEP) in childhood may influence the peak level of physical capability achieved in early adulthood, thereby affecting levels in later adulthood. We have undertaken a systematic review with meta-analyses to test the hypothesis that adverse childhood SEP is associated with lower levels of objectively measured physical capability in adulthood.

**Methods and Findings:**

Relevant studies published by May 2010 were identified through literature searches using EMBASE and MEDLINE. Unpublished results were obtained from study investigators. Results were provided by all study investigators in a standard format and pooled using random-effects meta-analyses. 19 studies were included in the review. Total sample sizes in meta-analyses ranged from N = 17,215 for chair rise time to N = 1,061,855 for grip strength. Although heterogeneity was detected, there was consistent evidence in age adjusted models that lower childhood SEP was associated with modest reductions in physical capability levels in adulthood: comparing the lowest with the highest childhood SEP there was a reduction in grip strength of 0.13 standard deviations (95% CI: 0.06, 0.21), a reduction in mean walking speed of 0.07 m/s (0.05, 0.10), an increase in mean chair rise time of 6% (4%, 8%) and an odds ratio of an inability to balance for 5s of 1.26 (1.02, 1.55). Adjustment for the potential mediating factors, adult SEP and body size attenuated associations greatly. However, despite this attenuation, for walking speed and chair rise time, there was still evidence of moderate associations.

**Conclusions:**

Policies targeting socioeconomic inequalities in childhood may have additional benefits in promoting the maintenance of independence in later life.

## Introduction

Maintenance of physical capability, that is an individual's ability to undertake the physical tasks of everyday living, is essential in older age. Grip strength, walking speed, time to rise from a chair and standing balance performance are simple, objective measures of physical capability levels that provide a marker of current health and predict subsequent health outcomes [1] including disability [2] and mortality [3], [4] in older populations.

Numerous studies have reported associations between socioeconomic position (SEP) and health in adulthood [5]–[7] with consistent evidence that the socioeconomically disadvantaged have higher chronic disease [8] and mortality rates [9]–[12] than the more advantaged. Evidence also indicates that socioeconomic disadvantage in childhood is associated with a range of adverse outcomes in adulthood [8], [13] often independent of adult SEP [11], [12]. Childhood SEP, through its association with a range of factors, including growth and early life nutrition, may influence the peak level of physical capability attained in early adulthood, thereby affecting levels later in life [14]. Adverse effects of SEP may also accumulate across the life course [15]. On the basis of such evidence it is argued that reducing health inequalities is a matter of fairness and social justice and, action to reduce health inequalities must start before birth and continue through life if the close links between early disadvantage and poor health are to be broken [16].

Poor adult SEP is associated with worse objectively measured physical capability levels [17], [18]; however, it is unclear whether this effect is also seen with childhood SEP independent of adult SEP. Such an association would have important implications for interventions aimed at improving the physical capability levels of older people and long term trends for ‘healthy ageing’ because of potential cohort effects and the compression of morbidity phenomenon [19]. To test the hypothesis that adverse childhood SEP is associated with lower levels of objectively measured physical capability in adulthood we have undertaken a systematic review and meta-analyses of both published and unpublished results.

## Methods

A systematic review of published literature was undertaken following the Meta-analysis of Observational Studies in Epidemiology (MOOSE) guidelines [20] and the PRISMA statement [21]. Unpublished results were then acquired through analysis of data from studies participating in the Healthy Ageing across the Life Course (HALCyon) collaboration (www.halcyon.ac.uk) and contact with other study investigators.

### Selection criteria

Eligible observational studies were those conducted on individual participants that examined the association between any indicator of childhood SEP (e.g. parental occupation or education) and at least one of four pre-specified objective measures of physical capability (grip strength, walking speed or get up and go test [22], chair rises and standing balance) in adulthood. Eligible study populations were community-dwelling adults aged 18 y or over at the time of physical capability measurement (full review protocol at www.halcyon.ac.uk).

### Literature search and data extraction

**Figure 1:**
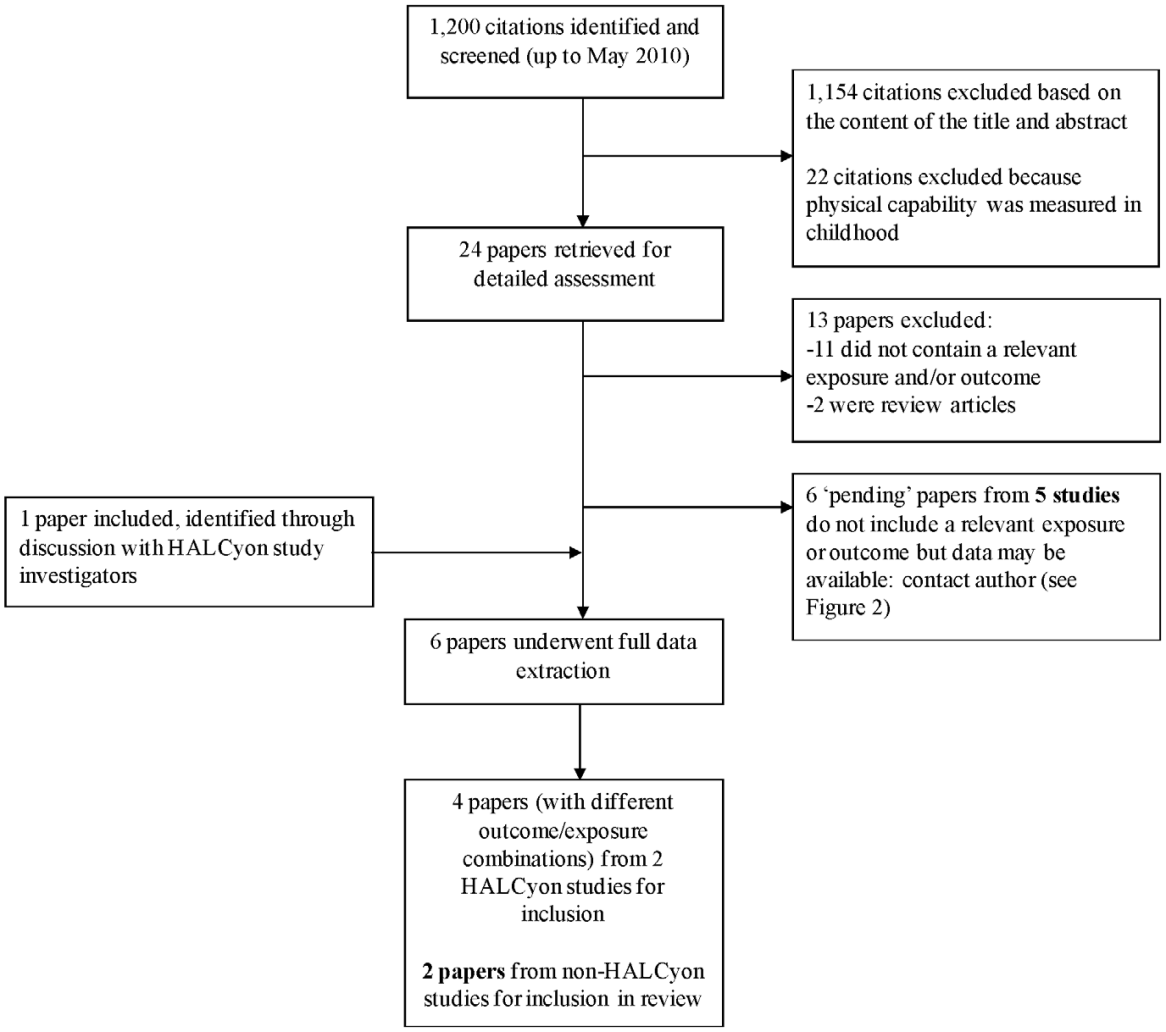
Flow diagram for identification of published studies.

Searches of the electronic databases MEDLINE and EMBASE (up to May 2010) were performed using text word search terms and explosion MeSH terms (Appendix S1) in any language (by KB). Searches were restricted to studies of humans. Duplicate records identified by title, authors, journal citation and date published, were removed. The abstracts of all 1,200 unique records identified were screened independently by two authors (KB and RC). The full text of 24 papers identified as potentially eligible were obtained with a final decision then made by consensus between KB and RC about eligibility. Of the 24 papers examined, five [23]–[27] reporting on three different studies, one of which participates in HALCyon [24]–[26], were eligible for inclusion. A sixth paper eligible for inclusion [28], also using data from a study participating in HALCyon, was identified through discussions with the study authors. A further six papers [29]–[34] identified during the screening process and reporting on five studies were classified as ‘pending’ because the papers did not present relevant results, but appropriate data might have been available. Figure 1 summarises this initial identification of studies.

Data from the six eligible papers were independently extracted by two authors (KB and RC) onto a standardised form. Information was extracted on associations of interest, the study population, baseline characteristics, details of the ascertainment of childhood SEP and physical capability measures, identification of potential confounders and methods of controlling for these. Any differences between the two sets of information extracted were resolved through discussion.

### Inclusion of unpublished results

#### HALCyon studies

We included data from eight of the nine UK cohort studies involved in the HALCyon collaboration. These are the Lothian Birth Cohort 1921 [35], the Hertfordshire Ageing Study [36], the Hertfordshire Cohort Study [37], the Caerphilly Prospective Study [38], the Aberdeen Birth Cohort 1936 [35), the Boyd Orr cohort [39], the English Longitudinal Study of Ageing [40] and the MRC National Survey of Health and Development [24]–[26]. Two of these studies [24]–[26], [28] had previously published on the associations of interest with findings from another two currently in press [41].

#### Other studies with relevant data

**Figure 2:**
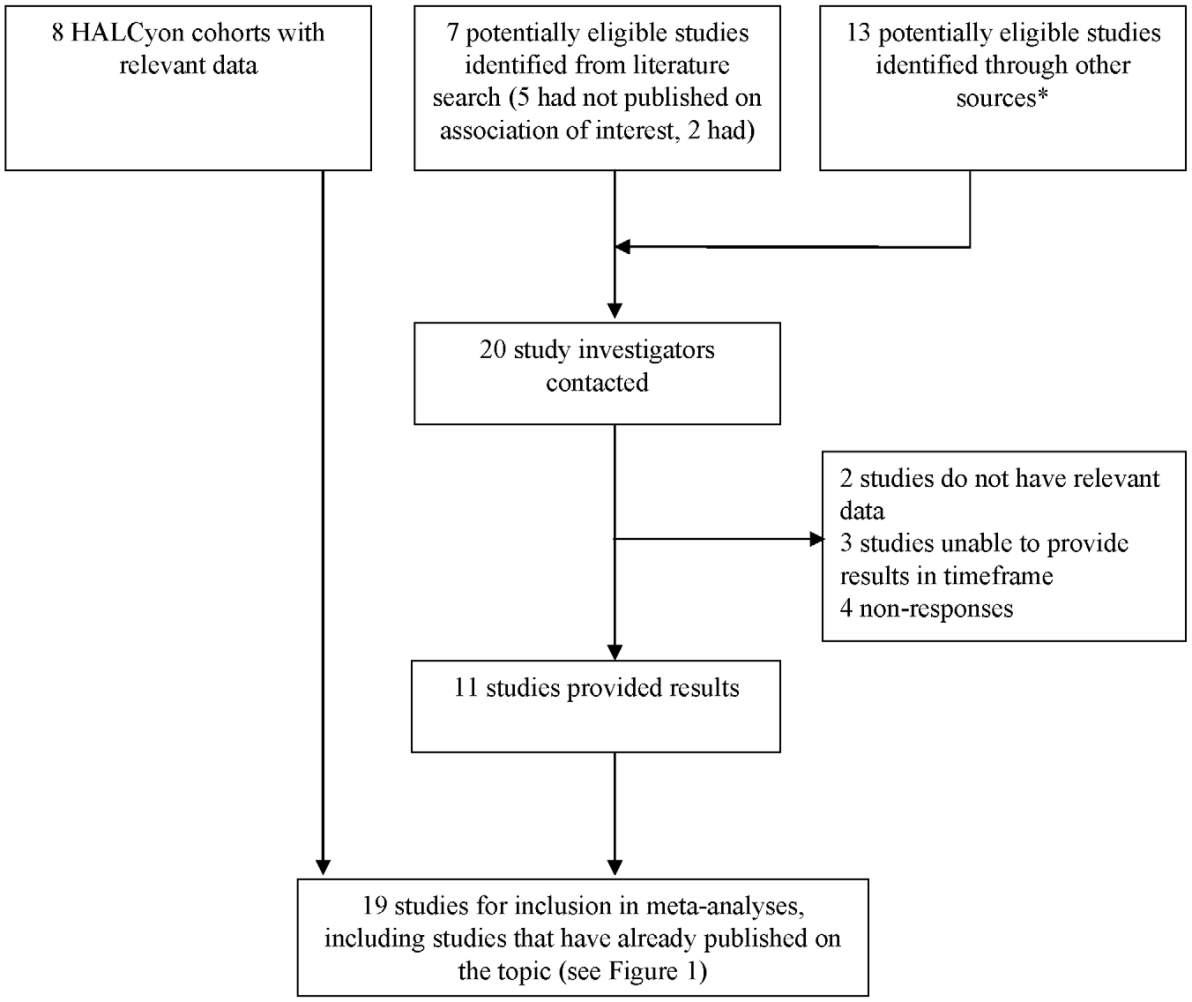
Flow diagram showing contact with authors and ascertainment of results for inclusion in review. * This included a review paper on longitudinal studies of ageing [55], relevant websites [56]–[58] and experts in the field of gerontology.

To ensure that all results for inclusion in meta-analyses were as comparable as possible, we contacted the corresponding authors of the other two studies [23], [27] identified from the electronic search as being eligible for inclusion. We also contacted the authors of the five ‘pending’ studies [29], [31]–[34] to ask whether they would be willing to provide results (Figure 2).

We identified an additional 13 studies [42]–[54] that we believed may have exposures and outcomes of interest, but had not published results from tests of these associations, by consulting a review paper on longitudinal studies of ageing [55], relevant websites [56]–[58] and asking experts in the field of gerontology. Investigators working on these studies were also contacted. In total, emails were sent to 20 study investigators. Responses to 16 of the 20 requests were received with eleven studies, including the two studies which had previously published on the associations, agreeing to provide results for inclusion in meta-analyses. The other five responses informed us of an inability to provide results (see Figure 2).

### Analyses requested from eligible studies

Using the eight HALCyon study datasets, we performed individual study analyses in a standard format for inclusion in meta-analyses. Investigators of the eleven other included studies [23], [27], [32], [43], [46], [48]–[50], [52]–[54] were asked to perform the same analyses and then complete standardised tables of results personalised for their study. These standard analyses involved testing the associations between *each* individual measure of childhood SEP and *each* measure of physical capability available, using sex-specific regression analyses. The indicators of childhood SEP and the physical capability measures were handled in the same way across studies.

#### Childhood SEP

Although the study protocol specified that any indicator of childhood SEP would be considered, we chose to focus in analyses on those measures most widely used across studies. These were father's occupation (usually assessed using the UK Registrar General's Social Classification system), childhood economic environment (usually assessed on a three point scale from good to poor or high to low), father's education and mother's education (both of which were usually based on a measure of length of time in education or highest level of education achieved). Each of these measures of childhood SEP was modelled as sex-specific ridit scores to enable direct comparison between cohorts (and SEP measures). The ridit scores take account of variation between studies in the methods of categorising SEP variables and in the proportions of people in different categories of a socioeconomic variable [59]. For each indicator of childhood SEP, after ordering the categories from highest to lowest, a score between 0 (highest SEP) and 1 (lowest SEP) was assigned to each category, based on the proportion of the population above the mid-point in that category. For example, if 10% of the population are in social class I, people in this group are represented by the range 0 to 0.1 and so are allocated the score 0.05 (i.e. divide 0.1 by 2 to obtain the value for the mid-point of the group). If 20% of the population are in the next highest group, social class II, then this social class is allocated a score 0.20 (0.1+0.2/2) and so on. Each of the outcomes can then be regressed on these ridit scores, with the regression coefficients representing the slope index of inequality (SII) for continuous outcomes and the relative index of inequality (RII) for binary outcomes. These are interpretable as comparing people of the lowest SEP (1) with people of the highest SEP (0), either in absolute (SII) or relative terms (RII).

#### Physical capability measures

Grip strength was analysed as an untransformed continuous variable with effect estimates converted to kg if strength had not been measured in kg. Timed walks and the get up and go test (which involves a chair rise followed by a timed walk) were converted to ‘walking speed’ in metres/second and analysed as untransformed continuous variables. Time to complete five chair rises was natural log transformed due to skewed distributions in most populations. Linear regression models were used to investigate associations of childhood SEP with grip strength, walking speed and log chair rise time. The regression coefficients for log chair rise time can be multiplied by 100 to represent percentage change in time [60]. Standing balance time could not be analysed as a continuous variable because a large proportion of participants in many studies achieved the maximum time of the test (generally around 30 seconds) and there was variation between studies in the methods of recording times. Standing balance time was thus dichotomised at a cut-point of five seconds, to identify those with the worst standing balance ability. Inability to balance on one leg for five seconds was used as the outcome event (coded as 1) in logistic regression models.

#### Adjustments

Three separate sets of adjustments were performed to test whether associations found were explained by the continuity of SEP from early life to adulthood and to control for body size which tends to be socioeconomically graded and is an important determinant of physical capability levels [25], [26]: (i) age; (ii) age and adult socioeconomic position (e.g. occupational class (of the head of household if available) and education); (iii) age, adult socioeconomic position and body size (height and weight or BMI).

### Meta-analyses

Random effects meta-analyses [61] were performed using the ‘metan’ command [62] in Stata version 11 [63] if sufficient results were available (i.e. more than three sets of comparable results). Random effects models were chosen *a priori* as we expected a large degree of heterogeneity between studies. Sex differences in the age adjusted associations were tested by meta-analyses of the within-study sex differences for each outcome. Where there was no evidence of sex differences within studies, estimates for men and women were included in all subsequent meta-analyses together. Summary estimates of effect were calculated for *each* different indicator of childhood SEP and its association with *each* physical capability measure. Meta-analyses were then used to calculate overall summary estimates of effect for the association of childhood SEP with each physical capability outcome using one childhood SEP estimate for each study and including all studies regardless of the indicator of SEP they had used. In studies with more than one measure of childhood SEP, the choice of indicator was based on the frequency of use across studies. Because it was the most frequently used indicator of childhood SEP among the included studies, paternal occupation was used if this measure was available, otherwise childhood economic circumstances was used and otherwise paternal education. Meta-analyses were first run on the age adjusted estimates, then repeated on the age and adult SEP adjusted estimates and finally on the age, adult SEP and body size adjusted estimates. Effect estimates from analyses of walking speed and timed get up and go speed were included in the same meta-analyses with the measure of walking speed used for those few studies which had measured both walking time and timed get up and go. For grip strength and walking speed, the sex-specific effect estimates were standardised by dividing the coefficients by the standard deviations (SDs). This takes into account variation in the distribution, in particular the SD, of physical capability outcomes between studies and between sexes within studies. For grip strength this also takes account of the differences between studies in the types of dynamometer used. Meta-analyses were performed on the unstandardised and standardised estimates, to examine whether standardisation reduced between-study heterogeneity.

The percentage of variation between studies that cannot be attributed to within-study variation was examined using the I^2^ value [64] and 95% CIs based on the statistical significance of Q [65]. Potential sources of heterogeneity were examined by stratifying meta-analyses by each of the following pre-specified factors: mean age of study participants (‘younger’ <60 y vs. ‘older’ ≥60 y), method of ascertaining childhood SEP (prospective vs. retrospective, because prospective studies are higher in the hierarchy of evidence) and study location (Europe vs. other, with the classification chosen pragmatically). Where there were sufficient sets of results (i.e. >10) meta-regression [66] was performed using the ‘metareg’ command [67] in Stata 11 [63] for pooled results for men and women in each study. We did not formally assess the quality of the included studies because, unlike randomised control trials, no validated quality criteria are available [68]. The main meta-analyses were re-run with each study removed in turn to test that no one study explained any heterogeneity found. We used funnel plots to assess bias (i.e. plots of study effect sizes against precision) and tested the symmetry of the funnel plots using Egger's test [69].

## Results

In total, 19 studies contributed results to this review; summaries of these studies are shown in Table S1. Most studies were of older populations with a median age at the time of physical capability assessment of 69 years (range 18 to 79 years, Table S1). The Swedish 1969/70 Conscription Cohort [32] was a subset of a study on the Swedish Military Service Conscription Register [49]. To avoid including the same study population in meta-analyses more than once, we included only the results from the Swedish Military Service Conscription Register in meta-analyses due to its greater sample size. The Survey on Health and Wellbeing of Elders (SABE) [23] contributes five data points per sex to the meta-analyses, because this was a multi-city study with heterogeneity in socioeconomic conditions between cities. In some cities, sample clusters were stratified in terms of geography, whereas in others the strata were defined both by geography and by aggregate indicators of socioeconomic conditions. Thus it was not considered appropriate by the SABE study investigators to group the participants from the different cities together when performing individual study analyses.

The total sample sizes included in the meta-analyses for each outcome are: N = 1,061,855 from 12 studies of grip strength; N = 20,770 from 13 studies of walking speed (10 which had assessed walking time and 3 which had used the get up and go test); N = 17,215 from 7 studies of chair rise time; and N = 22,156 from 11 studies of standing balance. There was no evidence of sex differences within studies for any outcome, so results for both sexes are presented in the same meta-analyses (p-values from meta-analyses of overall differences between sexes: grip strength [after standardisation of regression coefficients] p = 0.39; walking speed p = 0.71; chair rise time p = 0.97; standing balance p = 0.17). For walking speed, findings did not differ and heterogeneity between studies was not reduced when using standardised regression coefficients: therefore, for ease of interpretation, results from meta-analyses of unstandardised coefficients (m/s) are presented, however, for grip strength results from meta-analyses using standardised coefficients are presented.

### Age adjusted results

**Figure 3:**
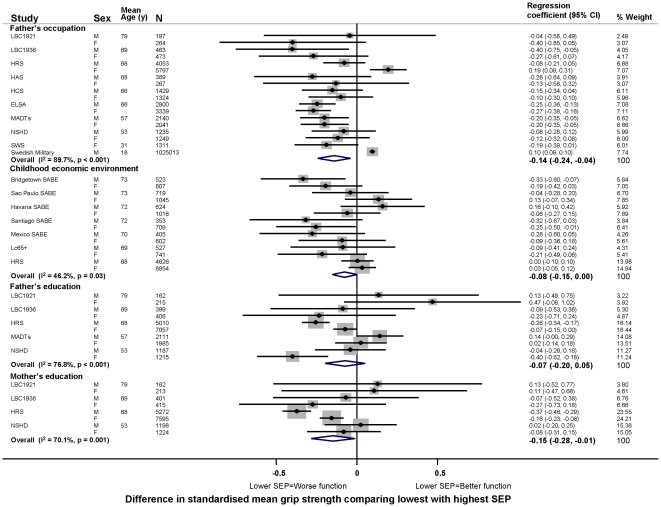
Age adjusted differences in mean standardised grip strength comparing lowest with highest childhood SEP. Footnotes: Please note that in the study of middle aged Danish twins (MADTs) major wage earner's occupation and education rather than father's occupation and education were assessed. Swedish 1969/70 Conscription Cohort was a subset of the study on the Swedish Military Service Conscription Register so has not been included in the meta-analysis. The results were: Swedish 1969/70 Conscription Cohort; 100% male; mean age 18.3 years; N = 42,365; the standardised estimate for father's occupation and grip strength was an increase of 0.24 SDs (95% CI: 0.21, 0.28). The abbreviations of study names for figures 3–6 are: ABC1921: Aberdeen Birth Cohort 1921; ABC1936: Aberdeen Birth Cohort 1936; Boyd Orr; CaPS: Caerphilly Prospective Study; ELSA: English Longitudinal Study of Ageing; HAS: Hertfordshire Aging Study; HCS: Hertfordshire Cohort Study; HRS: Health and Retirement Study; KLoSA: Korean Longitudinal Study of Ageing; LBC1921: Lothian Birth Cohort 1921; LBC1936: Lothian Birth Cohort 1936; Lc65+: Lausanne Cohort 65+; MADTs: The study of middle aged Danish twins; NSHD: MRC National Survey of Health and Development; PREHCO project: Puerto Rican Elderly Health Conditions project; SABE: Survey on Health and Wellbeing of Elders (conducted in: Bridgetown, Barbados; Havana, Cuba; Mexico City, Mexico; Santiago, Chile; Sao Paulo, Brazil); Swedish Military: Swedish Military Service Conscription Register; SWS: Southampton Women's Survey.

**Figure 4 pone-0015564-g004:**
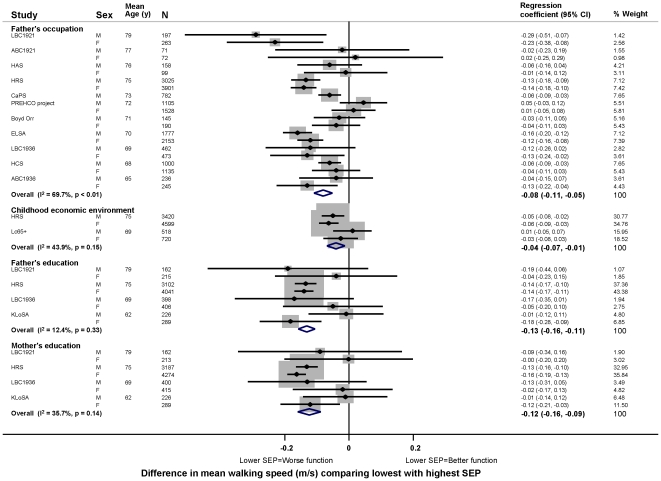
Age adjusted differences in mean walking speed (m/s) comparing lowest with highest childhood SEP.

**Figure 5 pone-0015564-g005:**
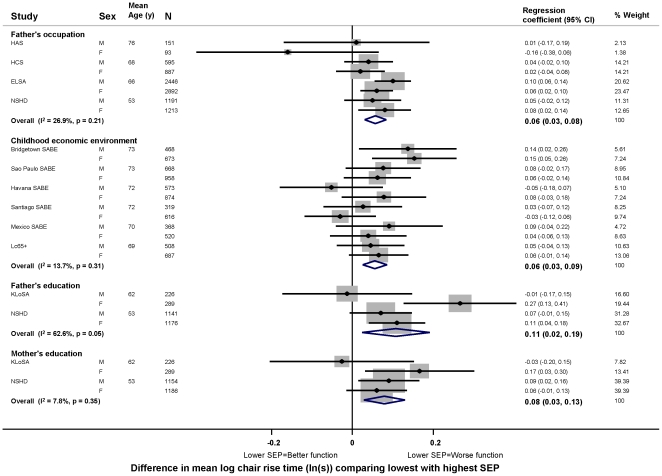
Age adjusted differences in mean chair rise time (ln(s)) comparing lowest with highest childhood SEP.

**Figure 6:**
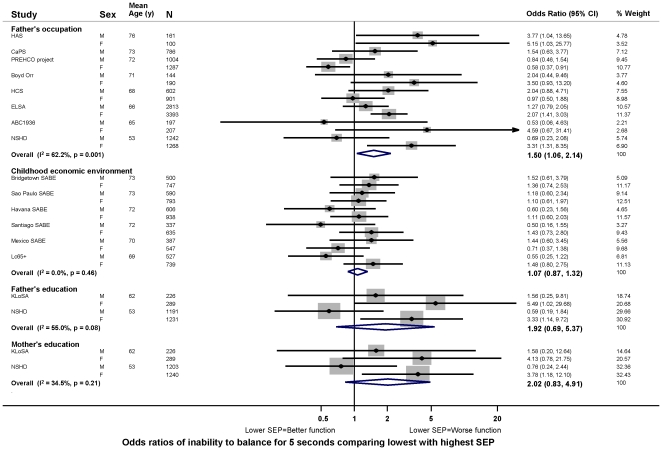
Age adjusted odds ratios of inability to balance for 5 seconds comparing lowest with highest childhood SEP.

In age adjusted models, there was evidence in the majority of studies that lower childhood SEP (i.e. less affluence), however it had been assessed, was associated with poorer physical capability levels, however this was assessed (Figures 3–6). For example, the overall summary age adjusted estimates of effect comparing the lowest with the highest father's occupational class were: −0.14 SDs for grip strength (95% CI: −0.24, −0.04; p = 0.01, N = 1,053,784) (Figure 3), with one SD in grip strength equal to approximately 9 kg in men and 6 kg in women; −0.08 m/s for walking speed (−0.11, −0.05; p<0.01, N = 19,017) (Figure 4); 6% for chair rise time (3%, 8%; p<0.01, N = 9,468) (Figure 5); and the odds ratio (OR) of inability to balance for five seconds was 1.50 (1.06, 2.14; p = 0.02, N = 14,295) (Figure 6). Although summary estimates of effect from meta-analyses of different indicators of childhood SEP are not directly comparable because of differences in the studies included in each comparison, there was a suggestion that the association of parental education with walking speed was stronger than the association of either father's occupation or childhood economic environment (Figure 4).

### Overall summary estimates

When combining different indicators of childhood SEP in the same meta-analyses (Table 1), the findings were similar to the results from meta-analyses which assessed each measure of childhood SEP separately (Figures 3–6). The overall summary age adjusted estimates of effect comparing the lowest with the highest childhood SEP were: −0.13 SDs for grip strength (95% CI: −0.21, −0.06; p<0.01, N = 1,061,855); −0.07 m/s for walking speed (−0.10, −0.05; p<0.01, N = 20,770) (−0.31 SDs for walking speed on the standardised scale; −0.42, −0.20); 6% for chair rise time (4%, 8%; p<0.01, N = 17,215); and the OR of inability to balance for 5 s was 1.26 (1.02, 1.55; p = 0.03, N = 22,156).

**Table 1:** Overall summary estimates of effect for the associations between childhood SEP and physical capability from random effects meta-analyses using ridit scores and comparing lowest with highest SEP.

Model*	Regression coefficient†	95% CI	p-value	I^2^	95% CI	p-value‡
**Grip strength (sd score)** (N = 1,061,855) (15 data points for men and 15 for women)						
1	−0.13	(−0.21, −0.06)	0.001	86.1%	(81.3, 89.7)	<0.001
2	−0.04	(−0.10, 0.02)	0.16	69.2%	(55.2, 78.8)	<0.001
3	−0.02	(−0.07, 0.04)	0.60	65.0%	(48.4, 76.2)	<0.001
**Walking speed (m/s)** (N = 20,770) (13 data points for men and 12 for women)						
1	−0.07	(−0.10, −0.05)	<0.001	72.1%	(58.4, 81.3)	<0.001
2	−0.02	(−0.04, −0.01)	0.004	23.6%	(0.0, 53.3)	0.14
3	−0.02	(−0.04, −0.001)	0.015	20.0%	(0.0, 51.1)	0.19
**Chair rises (ln(s))** § (N = 17,215) (11 data points for men and 11 for women)						
1	0.06	(0.04, 0.08)	<0.001	33.6%	(0.0, 60.4)	0.06
2	0.03	(0.01, 0.05)	0.01	19.8%	(0.0, 52.2)	0.20
3	0.03	(0.01, 0.05)	0.02	28.0%	(0.0, 57.3)	0.11
**Standing balance** (N = 22,156) (15 data points for men and 14 for women)						
1	1.26	(1.02, 1.55)	0.03	47.5%	(19.0, 66.0)	0.003
2	1.06	(0.86, 1.30)	0.60	41.5%	(8.9, 62.5)	0.01
3	1.02	(0.84, 1.24)	0.85	34.7%	(0.0, 58.5)	0.04

* Model 1: Age adjusted; Model 2: Age and adult SEP adjusted; Model 3: Age, adult SEP and body size adjusted.

† Mean difference in standard deviation score of grip strength; Mean difference in walking speed (m/s); Mean difference in natural log transformation of chair rise time (ln(s)); Odds ratio of inability to balance for 5 s for standing balance comparing lowest versus highest SEP based on ridit scores.

‡ *p*-values from Cochran's *Q* statistic.

§ The regression coefficients for chair rise time can be multiplied by 100 to represent percentage change in time [60].

Note: These models include estimates from studies for father's occupation if available, childhood economic environment if not and father's education if neither other measure of childhood SEP available.

### Adult SEP and body size adjusted results

After adjustment for adult SEP, associations were attenuated substantially (i.e. by 50 to 75%) for all outcomes (Table 1). For grip strength and standing balance, further attenuation occurred after additional adjustment for body size whereby associations were consistent with chance (−0.02 SDs for grip strength; 95% CI -0.07, 0.04; p = 0.59 and OR of inability to balance for 5 s 1.02; 0.84, 1.24; p = 0.85) (Table 1). However, for walking speed and chair rise time despite substantial attenuation there was still evidence of modest associations with childhood SEP in fully adjusted models: −0.02 m/s for walking speed (−0.04, −0.001; p = 0.02) (−0.08 SDs for walking speed on the standardised scale; −0.15, −0.01; p = 0.03) and 3% for chair rise time (1%, 5%; p = 0.02) (Table 1).

### Heterogeneity

There was evidence of substantial heterogeneity between studies in meta-analyses of grip strength I^2^ = 86.1% (95% CI: 81.3, 89.7) and walking speed I^2^ = 72.1% (58.4, 81.3) and moderate heterogeneity for chair rise time I^2^ = 33.6% (0.0, 60.4) and standing balance I^2^ = 47.5% (19.0, 66.0) (Table 1), with adjustment for adult SEP and body size reducing the heterogeneity between studies (Table 1). In stratified meta-analyses, there was no clear evidence that age or method of ascertaining SEP (pre-specified factors) explained the heterogeneity found (Table 2) and meta-regression analyses were not conducted on either of these factors because of limited power. In stratified meta-analyses for grip strength and standing balance, the associations found in age adjusted models were stronger in European studies than in studies from other parts of the world (Table 2). Meta-regression analyses provided further evidence of a difference in effect by study location for standing balance (OR for non-European compared to European = 0.58; 0.36, 0.94; p = 0.03) but there was no evidence of differences by location in meta-regression analyses of the other three outcomes (coefficients for non-European compared to European are: 0.05 SDs for grip strength; −0.12, 0.23, p = 0.51; 0.01 m/s for walking speed; −0.09, 0.12, p = 0.81; and 1% for chair rise time; −6%, 7%, p = 0.84). In most instances the removal of each individual study from the meta-analyses did not influence estimates of the level of heterogeneity or main findings greatly. The main findings remained the same even when the largest study (the Swedish Military Service Conscription Register, N = 1,025,013) which found an association in the opposite direction to most other studies for grip strength, was removed (results not shown) however the estimated level of heterogeneity between studies was lower when this study was removed with I^2^ reduced from 86.1% to 58.7%. The funnel plots (data not shown) and Egger test showed no evidence of small-study bias for walking speed (p = 0.60), chair rise time (p = 0.54) or standing balance (p = 0.24). However, for grip strength, the funnel plot was asymmetrical (p<0.001) although on further investigation this asymmetry was found to be attributable to the inclusion of the Swedish Military Service Conscription Register and when this study was removed from the plot there was no longer evidence of bias (p = 0.40).

**Table 2:** Overall age adjusted summary estimates of effect for the associations between childhood SEP and physical capability from random effects meta-analyses using ridit scores and comparing lowest with highest SEP stratified by age, method of ascertaining SEP and location.

Stratification		No. of data pointsM; F	Total N	Regression coefficient†	95% CI	p-value	I^2^	p-value‡
**Grip strength (sd score)**								
Age group (y)	<60	3;3	1,032,989	−0.11	(−0.27, 0.05)	0.18	89.1%	<0.001
	60+	12;12	28,866	−0.14	(−0.22, −0.06)	0.001	64.8%	<0.001
Ascertainment of SEP	Prospective	1;1	2,484	−0.10	(−0.24, 0.04)	0.16	0.0%	0.79
	Retrospective	14;14	1,059,371	−0.14	(−0.22, −0.05)	0.001	86.6%	<0.001
Location	Europe	9;9	1,045,202	−0.17	(−0.29, −0.06)	0.003	89.4%	<0.001
	Other	6;6	16,653	−0.08	(−0.19, 0.04)	0.19	68.8%	<0.001
**Walking speed (m/s)**								
Age group (y)	<60	0;0	0	N/A				
	60+	13;12	20,770	−0.08	(−0.10, −0.05)	<0.001	72.1%	<0.001
Ascertainment of SEP	Prospective	1;1	335	−0.04	(−0.09, 0.02)	0.21	0.0%	0.86
	Retrospective	12;11	20,435	−0.08	(−0.11, −0.05)	<0.001	73.6%	<0.001
Location	Europe	10;9	10,696	−0.07	(−0.10, −0.05)	<0.001	61.4%	<0.001
	Other	3;3	10,074	−0.07	(−0.14, 0.001)	0.05	86.5%	<0.001
**Chair rises (ln(s))** §								
Age group (y)	<60	1;1	2,404	0.07	(0.02, 0.11)	0.007	0.0%	0.54
	60+	10;10	14,811	0.06	(0.03, 0.08)	<0.001	39.1%	0.04
Ascertainment of SEP	Prospective	1;1	2,404	0.07	(0.02, 0.11)	0.007	0.0%	0.54
	Retrospective	10;10	14,811	0.06	(0.03, 0.08)	<0.001	39.1%	0.04
Location	Europe	5;5	10,663	0.06	(0.04, 0.08)	<0.001	7.4%	0.37
	Other	6;6	6,552	0.07	(0.02, 0.11)	0.003	49.7%	0.03
**Standing balance**								
Age group (y)	<60	1;1	2,510	1.56	(0.34, 7.23)	0.57	78.0%	0.03
	60+	14;13	19,646	1.24	(1.00, 1.52)	0.05	45.6%	0.01
Ascertainment of SEP	Prospective	2;2	2,844	2.00	(0.90, 4.45)	0.09	44.2%	0.15
	Retrospective	13;12	19,312	1.20	(0.97, 1.48)	0.09	46.5%	0.01
Location	Europe	8;7	13,270	1.60	(1.18, 2.15)	0.002	41.8%	0.05
	Other	7;7	8,886	1.00	(0.80, 1.26)	1.00	25.2%	0.18

† Mean difference in standard deviation score of grip strength; Mean difference in walking speed (m/s); Mean difference in natural log transformation of chair rise time (ln(s)); Odds ratio of inability to balance for 5 s for standing balance comparing lowest versus highest SEP based on ridit scores.

‡ *p*-values from Cochran's *Q* statistic.

§ The regression coefficients for chair rise time can be multiplied by 100 to represent percentage change in time [60]

M: Male; F: Female.

Note: These models include estimates from studies for father's occupation if available, childhood economic environment if not and father's education if neither other measure of childhood SEP available.

## Discussion

We found modest associations between indicators of childhood SEP and objectively measured physical capability levels in adulthood. People with lower SEP in childhood were more likely to have weaker grip strength, walk more slowly and perform less well in tests of chair rising and standing balance in later adulthood than people with higher childhood SEP, after adjustment for age. The associations of childhood SEP with walking speed and chair rise time were maintained, despite attenuations in effect size, after adjustment for indicators of adult SEP and current body size. However, the results from meta-analyses should be interpreted with some caution as there was evidence of unexplained heterogeneity between studies.

### Explanation of findings

Our finding of attenuations in effect size after adjustment for adult SEP suggests that associations between childhood SEP and physical capability levels could be partially explained by the tracking of SEP across life, with SEP in adulthood being a better predictor than childhood SEP. However, childhood SEP was measured by recall in all but two studies [39], [70] and would be expected to be more prone to measurement error than adult SEP which could dilute the size of effects estimated for childhood SEP. Furthermore, adjusting for adult SEP and adult body size could be considered an over-adjustment, if these factors lie on the causal pathway.

The associations of childhood SEP with walking speed and chair rise performance were maintained after adjustment for adult SEP, suggesting that the accumulation of adverse exposures over a lifetime may be a better model of the associations than one which considers only adult factors. There are several potential pathways that may link childhood SEP to adult physical capability. For example nutrition, motor development, physical activity and fitness in early life are socioeconomically graded and track into adulthood and such factors as these and others including stress and inflammation should be investigated further in future work. However, although prenatal growth, indexed by birth weight, is consistently related to adult grip strength [25], [44], [71] and is socioeconomically graded, in adjusted analyses childhood SEP and grip strength were not associated suggesting that this is one pathway unlikely to explain the observed associations.

### Possible sources of heterogeneity

Eleven of the nineteen studies included in this review are from the UK, in part because of the inclusion of the HALCyon cohorts. However, eligible studies have also been conducted in other European countries, [27], [32], [43], [49] the USA, [46], [48] Central and South America and the Caribbean [23] and Korea [53]. When comparing results by study location, most studies conducted in Europe used father's occupation as an indicator of SEP, whereas studies from other parts of the world used childhood economic environment or parental education. The differences found by study location may, therefore, be explained by differences in SEP indicator used.

There were differences between studies in the protocols followed for assessment of physical capability which could have contributed to the heterogeneity observed. A range of different handheld dynamometers [72] were used to measure grip strength (Table S1) with either the average or maximum value achieved over a set number of trials used in analyses. However, by using standardised regression coefficients in meta-analyses differences between studies in the types of dynamometer used were taken into account. Walking times were measured over different distances ranging from 8 feet (equivalent to 2.4 metres) [40] to 20 metres [43]. Walking and get up and go times were converted into speeds to ensure measures were more comparable across studies, despite differences in distance. However, participants may tackle a test differently depending on distance. Further, while in most studies participants were asked to walk at a normal pace, in a small number of studies [35], [52] participants were asked to walk as fast as possible. For chair rises, all participants were asked to perform five, except for the NSHD, where times to complete five rises were estimated from times to complete ten rises using sex-specific conversion factors derived from ELSA participants of a similar age who undertook both five and ten chair rises. Standing balance was always measured with eyes open, but other differences in the tests existed. Times were dichotomised to make comparisons across studies possible, but the categorisation may have produced a weaker measure of true balance ability than would have been achieved using a continuous measure.

A further possibility is that the heterogeneity between studies is real. It is plausible that the associations of childhood socioeconomic position with physical capability vary by study context including geographical location and birth period, whereby SEP in early life may play a more important role in some contexts than others.

### Strengths and limitations

Two main strengths of this systematic review are the inclusion of several objective measures of physical capability and the wide range of different studies. By following a strict protocol, testing *a priori* hypotheses and including many unpublished results, we hope to have minimised various sources of bias including selection and publication biases. A further strength is that by requesting that study authors perform their analyses in a standardised way, we have been able to limit the possibility that heterogeneity between studies is explained by variation in analytical methods, which may occur when using only published results.

There are also some potential limitations to this review. Firstly, ridit scores were used to model our main explanatory variables as it allows more valid comparisons of results across studies where the distribution of childhood SEP varies; however, by using this method, we are assuming that the relationship of childhood SEP with physical capability is linear. While this seems to be a reasonable assumption as many associations between SEP and health outcomes are linear [73]–[75], in some cohorts this assumption may be violated and this would lead to an underestimation of the size of association between childhood SEP and physical capability. However, there was little evidence of non-linearity in the HALCyon cohorts when this was investigated (results not shown). Secondly, the study participants included in analyses were selected on the basis of the availability of the outcome variable. People who have difficulty undertaking the physical capability tests [76] are, therefore, often excluded from analyses. This is a particular problem in older study populations as non-participation in physical capability tests is often found to be higher in subjects who have fallen in the previous year, use a walking aid, or have impaired activities of daily living [77]. We did not account for non-participation in analyses, except when considering standing balance where those people unable to undertake the test were included in the group classified as unable to balance for at least five seconds, therefore associations between adverse childhood SEP and physical capability in adulthood may have been underestimated. Attrition of the original samples (through non-response and death) is another potential limitation and may account for some differences between study populations found. Another potential limitation is that some studies adjusted for adult height and weight, whereas other studies adjusted for BMI and this may not remove confounding effects of height on physical capability. In addition there are other potential confounding factors, such as medication use and health status, which have not been included in these analyses, as it was decided that requesting further adjustments may lead to inconsistencies in adjustments across studies, but which may play some part in explaining the associations found.

We were unable to explain all the heterogeneity between studies and we may have failed to identify subgroup effects due to lack of power. Further, although we specified possible sources of heterogeneity *a priori*, the effects of the characteristics we investigated were potentially confounded by each other and by other factors. By examining only a small range of indicators of childhood SEP and being able to include only one indicator per study in our final set of analyses we may not have appropriately captured the aspects of the childhood economic environment which are most important in relation to physical capability, however, the indicators used are those which are most frequently measured across studies.

### Implications

This review demonstrates the impact of childhood SEP on physical capability levels in adulthood, which in turn are predictors of subsequent mortality in older community-dwelling populations [4]. To illustrate the potential impact of childhood SEP, we have used estimates from our previous meta-analysis [4] to predict how these associations translate into mortality differentials. A quartile change in walking speed is approximately one SD change (in terms of normal distributions). A quartile slower walking speed was associated with a mortality hazard ratio (HR) of 1.38 [4]. Our age adjusted estimate comparing the lowest with the highest childhood SEP was −0.31 SDs for walking speed. A 0.31 SD slower walking speed would be expected to have a HR of 1.11 i.e. an 11% increased hazard of death for those who are most deprived in childhood compared to those who are least deprived. The assumption of a linear quartile change representing an SD change is confirmed by the ilSIRENTE study [78], which considered walking speed as a continuous measure and was not included in the pooled estimate for the meta-analysis [4]. It should also be noted that the impact of these effects on physical dependency, quality of life, medical and social care are likely to be far greater. For example, life expectancy in the UK for people living in the poorest neighbourhoods is seven years shorter than for people living in the richest neighbourhoods but the difference in disability-free life expectancy is even more marked at seventeen years [16]. Thus people in poorer areas die sooner and spend more of their shorter lives with a disability [16]. Analyses of US Civil War veterans suggest that recent declines in disability rates were a continuation of declines in both chronic disease and disability occurring over the past century due to improved nutrition, sanitation, and education [79] which is consistent with our finding of a role for SEP in explaining variation in physical capability levels. If future improvements in life expectancy are to be matched with compression of morbidity [19], then policy makers must tackle the underlying causes behind social inequalities across the life course as well as implementing effective interventions for current older people.

### Conclusion

This systematic review provides evidence of modest associations between childhood SEP and physical capability levels in adulthood, although considerable heterogeneity between studies was observed. When considering methods of improving the physical capability levels of future populations of older adults, it is necessary to consider the long-term impact of childhood socioeconomic position and the role of socioeconomically graded risk factors in early life.

## Supporting Information

Table S1 **Characteristics of studies included in the review.**
(DOC)

Appendix S1 **Search strategy for systematic review of published literature on the association between childhood socioeconomic position and physical capability.**
(DOC)

## References

[1] Cooper R, Kuh D, Cooper C, Gale CR, Lawlor DA (2011). Objective measures of physical capability and subsequent health: a systematic review.. Age Ageing.

[2] Onder G, Penninx BW, Ferrucci L, Fried LP, Guralnik JM (2005). Measures of physical performance and risk for progressive and catastrophic disability: results from the Women's Health and Aging Study.. J Gerontol A Biol Sci Med Sci.

[3] Guralnik JM, Ferrucci L, Simonsick EM, Salive ME, Wallace RB (1995). Lower-extremity function in persons over the age of 70 years as a predictor of subsequent disability.. N Engl J Med.

[4] Cooper R, Kuh D, Hardy R, The Mortality Review Group, on behalf of the FALCon and HALCyon study teams (2010). Objectively measured physical capability levels and mortality: a systematic review and meta-analysis.. Br Med J.

[5] Kaplan GA, Lynch JW (1997). Whither studies on the socioeconomic foundations of population health?. Am J Public Health.

[6] Chandola T, Ferrie J, Sacker A, Marmot M (2007). Social inequalities in self reported health in early old age: follow-up of prospective cohort study.. Br Med J.

[7] Banks J, Marmot M, Oldfield Z, Smith JP (2006). Disease and disadvantage in the United States and in England.. JAMA.

[8] Galobardes B, Davey Smith G, Lynch JW (2006). Systematic review of the influence of childhood socioeconomic circumstances on risk for cardiovascular disease in adulthood.. Ann Epidemiol.

[9] Turrell G, Lynch JW, Leite C, Raghunathan T, Kaplan GA (2007). Socioeconomic disadvantage in childhood and across the life course and all-cause mortality and physical function in adulthood: evidence from the Alameda County Study.. J Epidemiol Community Health.

[10] Breeze E, Sloggett A, Fletcher A (1999). Socioeconomic and demographic predictors of mortality and institutional residence among middle aged and older people: results from the Longitudinal Study.. J Epidemiol Community Health.

[11] Davey Smith G, Hart C, Blane D, Gillis C, Hawthorne V (1997). Lifetime socioeconomic position and mortality: prospective observational study.. Br Med J.

[12] Lawlor DA, Sterne JA, Tynelius P, Davey Smith G, Rasmussen F (2006). Association of childhood socioeconomic position with cause-specific mortality in a prospective record linkage study of 1,839,384 individuals.. Am J Epidemiol.

[13] Galobardes B, Lynch JW, Davey Smith G (2004). Childhood socioeconomic circumstances and cause-specific mortality in adulthood: systematic review and interpretation.. Epidemiol Rev.

[14] Kuh D (2007). A life course approach to healthy aging, frailty, and capability.. J Gerontol A Biol Sci Med Sci.

[15] Ben-Shlomo Y, Kuh D (2002). A life course approach to chronic disease epidemiology: conceptual models, empirical challenges and interdisciplinary perspectives.. Int J Epidemiol.

[16] Fair society, healthy lives. The Marmot Review; 2010 10.1016/j.socscimed.2010.07.002 20685020

[17] Brunner E, Shipley M, Spencer V, Kivimaki M, Chandola T (2009). Social inequality in walking speed in early old age in the Whitehall II study.. J Gerontol A Biol Sci Med Sci.

[18] Hairi FM, Mackenbach JP, Andersen-Ranberg K, Avendano M (2010). Does socioeconomic status predict grip strength in older Europeans? Results from the SHARE study in non-institutionalized men and women aged 50+.. J Epidemiol Community Health.

[19] Fries JF (2000). Compression of morbidity in the elderly.. Vaccine.

[20] Stroup DF, Berlin JA, Morton SC, Olkin I, Williamson GD (2000). Meta-analysis of observational studies in epidemiology: a proposal for reporting.. JAMA.

[21] Moher D, Liberati A, Tetzlaff J, Altman DG (2009). Preferred reporting items for systematic reviews and meta-analyses: the PRISMA statement.. PLoS Med.

[22] Podsiadlo D, Richardson S (1991). The timed "Up & Go": a test of basic functional mobility for frail elderly persons.. J Am Geriatr Soc.

[23] Alvarado BE, Zunzunegui MV, Beland F, Bamvita JM (2008). Life course social and health conditions linked to frailty in Latin American older men and women.. J Gerontol Biol Sci Med Sci.

[24] Guralnik JM, Butterworth S, Wadsworth ME, Kuh D (2006). Childhood socioeconomic status predicts physical functioning a half century later.. J Gerontol A Biol Sci Med Sci.

[25] Kuh D, Bassey J, Hardy R, Aihie Sayer A, Wadsworth M (2002). Birth weight, childhood size, and muscle strength in adult life: evidence from a birth cohort study.. Am J Epidemiol.

[26] Kuh D, Hardy R, Butterworth S, Okell L, Richards M (2006). Developmental origins of midlife physical performance: evidence from a British birth cohort.. Am J Epidemiol.

[27] Osler M, McGue M, Christensen K (2007). Socioeconomic position and twins' health: A life-course analysis of 1266 pairs of middle-aged Danish twins.. Int J Epidemiol.

[28] Syddall H, Evandrou M, Cooper C, Aihie Sayer A (2009). Social inequalities in grip strength, physical function, and falls among community dwelling older men and women: findings from the Hertfordshire Cohort Study.. J Aging Health.

[29] Rasmussen F, Kark M, Tholin S, Karnehed N, Tynelius P (2006). The Swedish Young Male Twins Study: a resource for longitudinal research on risk factors for obesity and cardiovascular diseases.. Twin Res Hum Genet.

[30] Wolanski N, Siniarska A, Teter A, Antoszewska A (1992). The effect of culture and genotype on motor development of parents and their children.. Stud Hum Ecol.

[31] Siniarska A, Koziol-Kolodziejska R, Mantorska T (1992). Biological status of Jastarnia, Szczawnica and Kroscienko populations as an effect of adaptation to seaside and low mountain conditions.. Stud Hum Ecol.

[32] Theorell T, Svensson J, Knox S, Ahlborg B (1982). Blood pressure variations across areas in the greater Stockholm region: analysis of 74,000 18-year-old men.. Soc Sci Med.

[33] Buchman AS, Wilson RS, Boyle PA, Bienias JL, Bennett DA (2007). Grip strength and the risk of incident Alzheimer's disease.. Neuroepidemiology.

[34] Henneberg M, Brush G, Harrison GA (2001). Growth of specific muscle strength between 6 and 18 years in contrasting socioeconomic conditions.. Am J Phys Anthropol.

[35] Deary IJ, Whiteman MC, Starr JM, Whalley LJ, Fox HC (2004). The impact of childhood intelligence on later life: following up the Scottish mental surveys of 1932 and 1947.. J Pers Soc Psychol.

[36] Syddall HE, Simmonds SJ, Martin HJ, Watson C, Dennison EM (2010). Cohort profile: The Hertfordshire Ageing Study (HAS).. Int J Epidemiol.

[37] Syddall HE, Aihie Sayer A, Dennison EM, Martin HJ, Barker DJ (2005). Cohort profile: the Hertfordshire cohort study.. Int J Epidemiol.

[38] Caerphilly and Speedwell Collaborative Group (1984). Caerphilly and Speedwell collaborative heart disease studies.. J Epidemiol Community Health.

[39] Martin RM, Gunnell D, Pemberton J, Frankel S, Davey Smith G (2005). Cohort profile: The Boyd Orr cohort–an historical cohort study based on the 65 year follow-up of the Carnegie Survey of Diet and Health (1937-39).. Int J Epidemiol.

[40] Marmot M, Banks J, Blundell R, Lessof C, Nazroo J (2002). Health, wealth and lifestyles of the older population in England: The 2002 English Longitudinal Study of Ageing ..

[41] Birnie K, Martin RM, Gallacher J, Bayer A, Gunnell D (2010). Socioeconomic disadvantage from childhood to adulthood and locomotor function in old age: a lifecourse analysis of the Boyd Orr and Caerphilly prospective studies.. J Epidemiol Community Health In press.

[42] Rautio N, Heikkinen E, Ebrahim S (2005). Socio-economic position and its relationship to physical capacity among elderly people living in Jyvaskyla, Finland: five- and ten-year follow-up studies.. Soc Sci Med.

[43] Santos-Eggimann B, Karmaniola A, Seematter-Bagnoud L, Spagnoli J, Bula C (2008). The Lausanne cohort Lc65+: a population-based prospective study of the manifestations, determinants and outcomes of frailty.. BMC Geriatr.

[44] Ridgway CL, Ong KK, Tammelin T, Sharp SJ, Ekelund U (2009). Birth size, infant weight gain, and motor development influence adult physical performance.. Med Sci Sports Exerc.

[45] Zhang Y, Niu J, Kelly-Hayes M, Chaisson CE, Aliabadi P (2002). Prevalence of symptomatic hand osteoarthritis and its impact on functional status among the elderly: The Framingham Study.. Am J Epidemiol.

[46] Haas S (2008). Trajectories of functional health: the ‘long arm’ of childhood health and socioeconomic factors.. Soc Sci Med.

[47] Tyas SL, Snowdon DA, Desrosiers MF, Riley KP, Markesbery WR (2007). Healthy ageing in the Nun Study: definition and neuropathologic correlates.. Age Ageing.

[48] Monteverde M, Noronha K, Palloni A (2009). Effect of early conditions on disability among the elderly in Latin America and the Caribbean.. Popul Stud (Camb).

[49] Silventoinen K, Magnusson PK, Tynelius P, Batty GD, Rasmussen F (2009). Association of body size and muscle strength with incidence of coronary heart disease and cerebrovascular diseases: a population-based cohort study of one million Swedish men.. Int J Epidemiol.

[50] Inskip HM, Godfrey KM, Martin HJ, Simmonds SJ, Cooper C (2007). Size at birth and its relation to muscle strength in young adult women.. J Intern Med.

[51] Yliharsila H, Kajantie E, Osmond C, Forsen T, Barker DJ (2007). Birth size, adult body composition and muscle strength in later life.. Int J Obes.

[52] Deary IJ, Gow AJ, Taylor MD, Corley J, Brett C (2007). The Lothian Birth Cohort 1936: a study to examine influences on cognitive ageing from age 11 to age 70 and beyond.. BMC Geriatr.

[53] Jang SN, Kawachi I, Chang J, Boo K, Shin HG (2009). Marital status, gender, and depression: analysis of the baseline survey of the Korean Longitudinal Study of Ageing (KLoSA).. Soc Sci Med.

[54] Starr JM, Deary IJ, Lemmon H, Whalley LJ (2000). Mental ability age 11 years and health status age 77 years.. Age Ageing.

[55] Seematter-Bagnoud L, Santos-Eggimann B (2006). Population-based cohorts of the 50 s and over: a summary of worldwide previous and ongoing studies for research on health in ageing.. Eur J Ageing.

[56] National Centre for Social Research: http://www.natcen.ac.uk/elsa/docs/findings.htm Accessed January 2009

[57] National Institute on Aging: http://www.nia.nih.gov/ResearchInformation/ScientificResources/LongitudinalStudiesAllCurrent.htm Accessed 19 February 2009

[58] Integrative Analysis of Longitudinal Studies on Aging: http://www.ialsa.org Accessed January 2009

[59] Mackenbach JP, Kunst AE (1997). Measuring the magnitude of socio-economic inequalities in health: an overview of available measures illustrated with two examples from Europe.. Soc Sci Med.

[60] Cole TJ (2000). Sympercents: symmetric percentage differences on the 100 log(e) scale simplify the presentation of log transformed data.. Stat Med.

[61] DerSimonian R, Laird N (1986). Meta-analysis in clinical trials.. Control Clin Trials.

[62] Harris R, Deeks J, Altman DG, Bradburn MJ, Harbord R (2008). Metan: fixed and random effects meta-analysis.. Stata J.

[63] StataCorp Stata: Release 11.. Statistical Software.

[64] Higgins JP, Thompson SG, Deeks JJ, Altman DG (2003). Measuring inconsistency in meta-analyses.. Br Med J.

[65] Higgins JP, Thompson SG (2002). Quantifying heterogeneity in a meta-analysis.. Stat Med.

[66] Thompson SG, Sharp SJ (1999). Explaining heterogeneity in meta-analysis: a comparison of methods.. Stat Med.

[67] Harbord RM, Higgins JPT (2008). Meta-regression in Stata.. Stata J.

[68] Juni P, Witschi A, Bloch R, Egger M (1999). The hazards of scoring the quality of clinical trials for meta-analysis.. JAMA.

[69] Egger M, Davey Smith G, Schneider M, Minder C (1997). Bias in meta-analysis detected by a simple, graphical test.. Br Med J.

[70] Wadsworth M, Kuh D, Richards M, Hardy R (2006). Cohort Profile: The 1946 National Birth Cohort (MRC National Survey of Health and Development).. Int J Epidemiol.

[71] Aihie Sayer A, Syddall HE, Gilbody HJ, Dennison EM, Cooper C (2004). Does sarcopenia originate in early life? Findings from the Hertfordshire cohort study.. J Gerontol A Biol Sci Med Sci.

[72] Bassey EJ, Harries UJ (1993). Normal values for handgrip strength in 920 men and women aged over 65 years, and longitudinal changes over 4 years in 620 survivors.. Clin Sci (Lond).

[73] Osler M, Madsen M, Nybo Andersen AM, Avlund K, McGue M (2009). Do childhood and adult socioeconomic circumstances influence health and physical function in middle-age?. Soc Sci Med.

[74] Lawlor DA, Davey Smith G, Ebrahim S (2004). Association between childhood socioeconomic status and coronary heart disease risk among postmenopausal women: findings from the British Women's Heart and Health Study.. Am J Public Health.

[75] Frankel S, Davey Smith G, Gunnell D (1999). Childhood socioeconomic position and adult cardiovascular mortality: the Boyd Orr Cohort.. Am J Epidemiol.

[76] Rockwood K, Awalt E, Carver D, MacKnight C (2000). Feasibility and measurement properties of the functional reach and the timed up and go tests in the Canadian study of health and aging.. J Gerontol A Biol Sci Med Sci.

[77] Lin MR, Hwang HF, Hu MH, Wu HD, Wang YW (2004). Psychometric comparisons of the timed up and go, one-leg stand, functional reach, and Tinetti balance measures in community-dwelling older people.. J Am Geriatr Soc.

[78] Cesari M, Onder G, Zamboni V, Manini T, Shorr RI (2008). Physical function and self-rated health status as predictors of mortality: results from longitudinal analysis in the ilSIRENTE study.. BMC Geriatr.

[79] Manton KG (2008). Recent declines in chronic disability in the elderly U.S. population: risk factors and future dynamics.. Annu Rev Public Health.

