# Supplementary material for: Childhood Socioeconomic Position and Objectively Measured Physical Capability Levels in Adulthood: A Systematic Review and Meta-Analysis
Source: PLoS One. 2011 Jan 26;6(1):e15564. doi: 10.1371/journal.pone.0015564 (PMC3027621; doi:10.1371/journal.pone.0015564)
Supplement: Table S1 — Characteristics of studies included in the review. (DOC) [file pone.0015564.s001.doc]

Table S1: Characteristics of studies included in the review

| **Study name and country** | **Source^*^** | **Characteristics of study population:**  **-Mean (SD) age (y) at performance test; range**  **-% female** | **Measures of childhood socioeconomic position^†^** | **Measures of physical capability examined and summary statistics for men; women:**  **mean (SD) or median (IQR) or n (%)^‡^** |
| --- | --- | --- | --- | --- |
| Aberdeen Birth Cohort 1921 (ABC1921), UK [54] | Au | 76.9 (0.4); 75.9-77.8  - 50.3% | Father’s occupation (R) | **Walking speed**  0.8 (0.2); 1.0 (0.3) |
| Aberdeen Birth Cohort 1936 (ABC1936), UK [35] | H | -64.7 (0.98); 62-68  -51.2% | Father’s occupation  (R) | **Walking speed**  1.3 (0.3); 1.2 (0.2)  **Standing balance**  12 (6.1%); 16 (7.7%) |
| Boyd Orr cohort, UK [39] | H | -70.8 (4.4); 64-82  -56.9% | Father’s occupation (P) | **TUG speed**  0.6 (0.1); 0.6 (0.2)  **Standing balance**  27 (18.8%); 43 (22.6%) |
| Caerphilly Prospective Study (CaPS), UK [38] | H | -75.3 (4.3); 66-86  -0% | Father’s occupation (R) | **TUG speed**  0.6 (0.1); N/A  **Standing balance**  204 (26.0%); N/A |
| English Longitudinal Study of Ageing (ELSA), UK [40] | H | -Walking speed:70.5 (7.5); 60-90+; Other measures: 65.9 (9.3); 52-90+  -54.7% | Father’s occupation (R) | **Grip strength^§^**  40.8 (9.6); 24.5 (6.4)  **Walking speed**  0.9 (0.3); 0.9 (0.3)  **Chair rises**  10.6 (8.7-13.1); 11.0 (8.9-13.6)  **Standing balance**  282 (10.0%); 530 (15.6%) |
| Health and Retirement Study (HRS), USA [46] | Au | For grip strength:  - 74.5 (7.1); 65-104  - 57.3%  For walking speed:  - 68.3 (10.5); 28-104  - 58.7% | Father’s occupation  Father’s education  Mother’s education  Childhood economic environment (R) | **Grip strength^§^**  39.0 (9.4); 24.1 (6.8)  **Walking speed**  0.9 (0.3); 0.8 (0.3) |
| Hertfordshire Ageing Study (HAS), UK [36] | H | -67.5 (2.3); 63-73  -40.7% | Father’s occupation (R) | **Grip strength^§^**  38.3 (7.2); 22.5 (5.1)  **Walking speed**  0.9 (0.2); 0.8 (0.2)  **TUG speed**  0.5 (0.1); 0.5 (0.1)  **Chair rises**  18.3 (15.6-21.9); 19.6 (17.0-24.1)  **Standing balance**  49 (30.4%); 36 (36.0%) |
| Hertfordshire Cohort Study (HCS), UK [28,37] | H/P | -66.2 (2.2); 59-73  -48.1% | Father’s occupation (R) | **Grip strength^§^**  44.0 (7.6); 26.5 (5.7)  **Walking speed**  0.9 (0.2); 0.9 (0.3)  **TUG speed**  0.6 (0.1); 0.6 (0.1)  **Chair rises**  15.1 (13.2-17.1); 17.2 (14.7-20.4)  **Standing balance**  89 (14.8%); 150 (16.7%) |
| Korean Longitudinal Study of Ageing (KLoSA), Korea [53] | Au | -62.0 (10.0); 45-93  -56.1% | Father’s education  Mother’s education (R) | **Walking speed**  1.0 (0.3); 0.9 (0.3)  **Chair rises**  10.7 (8.4-13.0); 11.6 (9.1-14.2)  **Standing balance**  22 (9.7%); 30 (10.4%) |
| Lausanne Cohort 65+ (Lc65+), Switzerland [43] | Au | -69.0 (1.5); 66-71  -58.4% | Childhood economic environment (R) | **Grip strength^§^**  40.2 (7.8); 23.7 (4.8)  **Walking speed**  1.2 (0.2); 1.1 (0.2)  **Chair rises**  10.6 (9.1-12.3); 11.5 (9.9-13.6)  **Standing balance**  115 (21.8%); 222 (30.0%) |
| Lothian Birth Cohort 1921 (LBC1921), UK [35] | H | -79.1 (0.6); 77-80  -57.3% | Father’s occupation  Father’s education  Mother’s education (R) | **Grip strength^§^**  34.9 (7.6); 20.6 (4.3)  **Walking speed**  1.5 (0.4); 1.3 (0.3) |
| Lothian Birth Cohort 1936 (LBC1936), UK [52] | Au | -69.5 (0.8); 67-71  -50.5% | Father’s occupation  Father’s education  Mother’s education (R) | **Grip strength^§^**  37.7 (7.5); 21.8 (5.1)  **Walking speed**  1.8 (0.4); 1.6 (0.3) |
| MRC National Survey of Health and Development, 1946 British birth cohort (NSHD), UK [24-26] | H/P | -53 (0); 53-53  -50.6% | Father’s occupation  Father’s education  Mother’s education (P) | **Grip strength^§^**  47.7 (12.3); 27.8 (8.0)  **Chair rises**  9.7 (7.9-11.6); 9.4 (8.1-11.6)  **Standing balance**  41 (3.3%); 64 (5.1%) |
| Puerto Rican Elderly Health Conditions project (PREHCO), Puerto Rico [48] | Au | -72.5 (9.0); 60-107  -58.0% | Father’s occupation (R) | **TUG speed**  0.5 (0.3); 0.5 (0.4)  **Standing balance**  184 (18.3%); 347 (27.0%) |
| Southampton Women’s Survey (SWS), UK [50] | Au | -30.7 (3.7); 20-40  -100% | Father’s occupation (R) | **Grip strength^§^**  N/A ; 32.2 (6.0) |
| Study of Middle Aged Danish twins (MADTs), Denmark [27] | Au/P | -56.9 (6.3); 45-68  -48.8 | Education and occupation of the major wage earner (R) | **Grip strength^§^**  47.8 (8.4); 27.7 (5.8) |
| Survey on Health and Wellbeing of Elders (SABE) - Bridgetown, Barbados [23] | Au/P | -72.6 (8.2); 60-97  -60.7% | Childhood economic environment (R) | **Grip strength^§^**  30.7 (8.8); 19.7 (6.0)  **Chair rises**  10 (9-15); 12 (10-15)  **Standing balance**  101 (20.2%); 246 (32.9%) |
| Survey on Health and Wellbeing of Elders (SABE) - Havana, Cuba [23] | Au/P | -72.0 (8.9); 60-102  -62.0% | Childhood economic environment (R) | **Grip strength^§^**  31.7 (9.1); 17.3 (5.8)  **Chair rises**  12 (10-16); 14 (11-19)  **Standing balance**  77 (12.7%); 202 (21.5%) |
| Survey on Health and Wellbeing of Elders (SABE) – Mexico City, Mexico [23] | Au/P | -69.9 (7.8); 60-98  -60.0% | Childhood economic environment (R) | **Grip strength^§^**  30.4 (7.1); 19.0 (5.1)  **Chair rises**  9 (7-11); 11 (9-13)  **Standing balance**  128 (33.1%); 279 (51.0%) |
| Survey on Health and Wellbeing of Elders (SABE) - Santiago, Chile [23] | Au/P | -71.6 (8.0); 60-99  -67.0% | Childhood economic environment (R) | **Grip strength^§^**  33.0 (8.6); 18.8 (5.8)  **Chair rises**  10 (8-11); 11 (9-14)  **Standing balance**  62 (18.4%); 231 (36.4%) |
| Survey on Health and Wellbeing of Elders (SABE) - Sao Paulo, Brazil [23] | Au/P | -73.3 (8.5); 60-100  -59.2% | Childhood economic environment (R) | **Grip strength^§^**  30.8 (8.5); 19.3 (5.5)  **Chair rises**  12 (10-14); 13 (10-16)  **Standing balance**  200 (33.9%); 323 (40.7%) |
| Swedish Military Service Conscription Register, Sweden [49] | Au | -18.3 (0.6); 16-26  -0% | Father’s occupation (R) | **Grip strength^§^**  62.7 (9.9); N/A |
| Swedish 1969/70 Conscription Cohort, Sweden [32] | Au | -18.3 (0.6); 18-20  -0% | Father’s occupation (R) | **Grip strength^§^**  61.6 (9.8); N/A |

* Source of results for inclusion in review

H: Part of the HALCyon collaboration

H/P: Part of the HALCyon collaboration and results from published paper

Au: Results provided by author, not available in published paper

Au/P: Results available in published paper and additional results also provided for review by author

† P=prospective ascertainment; R=retrospective ascertainment

‡ Grip strength in kg; Walking speed in metres/second; TUG speed = walking speed calculated using time for timed get up and go test; Chair rises in time (s) to complete 5 rises; Standing balance = n(%) unable to balance for 5s

§ Grip strength measured using: Smedley handgrip dynamometers in ELSA, HRS and MADTs; Jamar hydraulic dynamometers in HCS and SWS; Jamar or North Coast hydraulic dynamometers in LBC1921 and LBC1936; Harpenden handgrip dynamometers in HAS; Baseline hydraulic hand dynamometers in Lc65+; Nottingham electronic handgrip dynamometers in NSHD; unknown models of dynamometer in SABE and the Swedish Military studies (the latter because the study protocol was not released to study investigators by the Swedish army)
