# Supplementary material for: Childhood Socioeconomic Position and Objectively Measured Physical Capability Levels in Adulthood: A Systematic Review and Meta-Analysis
Source: PLoS One. 2011 Jan 26;6(1):e15564. doi: 10.1371/journal.pone.0015564 (PMC3027621; doi:10.1371/journal.pone.0015564)
Supplement: Appendix S1 — Search strategy for systematic review of published literature on the association between childhood socioeconomic position and physical capability. (DOC) [file pone.0015564.s002.doc]

**Appendix S1: Search strategy for systematic review of published literature on the association between childhood socioeconomic position and physical capability**

|  | **Search** | **Results** |
| --- | --- | --- |
| 1 | exp Gait/ | 21646 |
| 2 | gait speed.tw. | 1121 |
| 3 | “get up and go”.tw. | 179 |
| 4 | Locomotion/ and walking/ | 1648 |
| 5 | walk speed.tw. | 35 |
| 6 | (chair adj3 ris$).tw. | 599 |
| 7 | balance.tw. | 158051 |
| 8 | flamingo stand.tw. | 1 |
| 9 | postural control.tw. | 3284 |
| 10 | exp Muscle strength/ | 24063 |
| 11 | (muscle adj3 strength).tw. | 15378 |
| 12 | exp Hand strength/ | 9396 |
| 13 | grip strength.tw. | 7079 |
| 14 | handgrip strength.tw. | 676 |
| 15 | hand strength.tw. | 278 |
| 16 | physical performance.tw. | 4883 |
| 17 | exp Socioeconomic Factors/ | 294425 |
| 18 | socio economic factors.tw. | 1681 |
| 19 | socioeconomic factors.tw. | 4331 |
| 20 | (socioeconomic adj3 conditions).tw. | 2202 |
| 21 | (socio economic adj3 conditions).tw. | 1016 |
| 22 | (socio economic adj3 disadvant$).tw. | 222 |
| 23 | (socioeconomic adj3 disadvant$).tw. | 565 |
| 24 | (socio economic adj3 position).tw. | 297 |
| 25 | (socioeconomic adj3 position).tw. | 1168 |
| 26 | socio economic status.tw. | 6204 |
| 27 | socioeconomic status.tw. | 22404 |
| 28 | SES.tw. | 7167 |
| 29 | exp Social Class/ | 30985 |
| 30 | social class.tw. | 10270 |
| 31 | exp Social Conditions/ | 42166 |
| 32 | social conditions.tw. | 2227 |
| 33 | social circumstances.tw. | 1150 |
| 34 | exp Occupation/ | 85029 |
| 35 | occupation.tw. | 24740 |
| 36 | exp Employment/ | 54009 |
| 37 | employment.tw. | 43367 |
| 38 | manual.tw. | 63743 |
| 39 | Exp Income/ | 55532 |
| 40 | income.tw. | 53601 |
| 41 | exp Education/ | 759978 |
| 42 | education.tw. | 292522 |
| 43 | exp Social Mobility/ | 7551 |
| 44 | social mobil$.tw. | 626 |
| 45 | exp Poverty/ | 28679 |
| 46 | poverty.tw. | 14834 |
| 47 | deprivation.tw. | 57270 |
| 48 | exp Life Change Events/ | 23970 |
| 49 | lifecourse.tw. | 193 |
| 50 | life course.tw. | 2464 |
| 51 | early life.tw. | 9362 |
| 52 | childh$.tw. | 202671 |
| 53 | exp Child/ | 1887714 |
| 54 | (father$ adj3 occupation$).tw. | 635 |
| 55 | (mother$ adj3 occupation$).tw. | 389 |
| 56 | (parent$ adj3 occupation$).tw. | 1190 |
| 57 | (father$ adj3 social class$).tw. | 198 |
| 58 | (mother$ adj3 social class$).tw. | 146 |
| 59 | (parent$ adj3 social class$).tw. | 337 |
| 60 | (father$ adj3 education$).tw. | 806 |
| 61 | (mother$ adj3 education$).tw. | 2728 |
| 62 | (parent$ adj3 education$).tw. | 6207 |
| 63 | (father$ adj3 manual$).tw. | 62 |
| 64 | (mother$ adj3 manual$).tw. | 27 |
| 65 | (parent$ adj3 manual$).tw. | 69 |
| 66 | (father$ adj3 incom$).tw. | 116 |
| 67 | (mother$ adj3 incom$).tw. | 1112 |
| 68 | (parent$ adj3 incom$).tw. | 1207 |
| 69 | (child$ adj3 social class$).tw. | 525 |
|  | *“Or” for the physical performance measures:* |  |
| 70 | 1 or 2 or 3 or 4 or 5 or 6 or 7or 8 or 9 or 10 or 11 or 12 or 13 or 14 or 15 or 16 | 222436 |
|  | *“Or” for the socioeconomic position measures:* |  |
| 71 | 17 or 18 or 19 or 20 or 21 or 22 or 23 or 24 or 25 or 26 or 27 or 28 or 29 or 30 or 31 or 32 or 33 or 34 or 35 or 36 or 37 or 38 or 39 or 40 or 41 or 42 or 43 or 44 or 45 or 46 or 47 | 1333876 |
|  | *“Or” for the lifecourse/childhood:* |  |
| 72 | 48 or 49 or 50 or 51 or 52 or 53 | 1979281 |
|  | *“And” to combine social class with lifecourse/childhood:* |  |
| 73 | 70 and 71 | 157127 |
|  | *“Or” for the other way of defining childhood socioeconomic position:* | |
| 74 | 54 or 55 or 56 or 57 or 58 or 59 or 60 or 61 or 62 or 63 or 64 or 65 or 66 or 67 or 68 or 69 | 13605 |
|  | *“Or” for the two ways of gaining childhood socioeconomic position:* | |
| 75 | 73 or 74 | 163188 |
|  | *“And” to combine physical performance with childhood socioeconomic position:* | |
| 76 | 70 and 75 | 1367 |
| 77 | limit 76 to humans | 1328 |
| 78 | remove duplicates from 77 | 1087 |

Exp: explode

Tw: text word

$ allows variation in word ending

adj3: adjacent three words

Note: Results displayed are for joint search from Medline 1950 to February 2009 and EMBASE to February 2009, this has been updated to May 2010.
